# Apoptosis and autophagy promote *Babesia microti* infection in tick midguts: insights from transcriptomic and functional RNAi studies

**DOI:** 10.3389/fmicb.2025.1632974

**Published:** 2025-09-19

**Authors:** Songqin Chen, Shanming Hu, Fengjun Gong, Haotian Zhu, Yongzhi Zhou, Jie Cao, Houshuang Zhang, Yanan Wang, Jinlin Zhou

**Affiliations:** ^1^Shanghai Veterinary Research Institute, Chinese Academy of Agricultural Sciences, Shanghai, China; ^2^College of Animal Science and Technology, Anhui Agricultural University, Hefei, China

**Keywords:** *B. microti*, tick, midgut, RNA-seq, apoptosis, autophagy

## Abstract

**Introduction:**

Ticks are the primary vectors of *Babesia* sp, with the midgut as the initial site of pathogen invasion following blood feeding. Elucidating the molecular interactions between tick midguts and *Babesia* is essential for developing targeted strategies to control tick-borne babesiosis. However, studies in this field remain limited.

**Methods:**

To investigate tick-pathogen interactions, we employed RNA-seq to profile gene expression, and qRT-PCR served to validate key findings. Apoptosis and autophagy were assessed via TUNEL staining and Transmission Electron Microscopy (TEM). Furthermore, RNA interference (RNAi) and pharmacological modulation were employed to evaluate the impact of ticks on pathogen load.

**Results:**

Our RNA-seq analysis identified 540 and 569 Differentially Expressed Genes (DEGs) in infected midguts at 0 and 4 d post-engorgement, respectively. These DEGs were enriched in pathways related to metabolic processes, immunity, and cellular processes. To clarify the functional relevance of these findings, the roles of apoptosis and autophagy during infection were further evaluated. Quantitative Real-Time PCR (qRT-PCR) analysis revealed significant upregulation of apoptosis-related genes (*caspase-7, caspase-8*, and *caspase-9*) and autophagy genes (*ATG5, ATG8, and ATG12*) in response to *B. microti* infection. TUNEL assay and Transmission Electron Microscopy (TEM) analysis demonstrated that *B. microti* infection significantly induced apoptosis and autophagosome formation in tick midgut tissues. Functional assays demonstrated that RNA interference (RNAi)-mediated knockdown of caspase-7, caspase-9, and ATG5 significantly reduced the *burden of B. microti*. Conversely, pharmacological induction of autophagy using rapamycin increased *B. microti* load, whereas inhibition with 3-methyladenine (3-MA) decreased *B. microti* load.

**Discussion:**

These findings underscore the critical roles of apoptosis and autophagy in facilitating *B. microti* infection within tick midguts, highlighting these pathways as potential molecular targets for disrupting the transmission of tick-borne *Babesia* infections.

## 1 Introduction

The Asian longhorned tick (*Haemaphysalis longicornis*) is an invasive ectoparasitic arthropod of significant concern to public health and agriculture. Originally endemic to East Asia, Australia, and New Zealand, this highly adaptable species has successfully colonized persistent, self-sustaining populations in at least nineteen states across the eastern United States, underscoring its notable ecological plasticity. This adaptability is largely attributed to biological traits, such as parthenogenetic reproduction and a broad thermal tolerance range (-4 to 40 C) (Beard et al., 2018; Rainey et al., 2018; Rochlin, 2019; Tanne, 2018). As a generalist hematophagous vector, *H. longicornis* exhibits a broad host specificity, parasitizing over forty species of mammals and birds. It serves as a competent vector for numerous pathogens, including Severe Fever With Thrombocytopenia Syndrome Virus (SFTSV), Tick-Borne Encephalitis Virus (TBEV), *Anaplasma phagocytophilum, Borrelia burgdorferi, Babesia* spp., *Theileria orientalis, Rickettsia* spp., and *Ehrlichia* spp (Dobler et al., 2012; Kang et al., 2016; Rosenberg et al., 2018; Zhao et al., 2021; Zhuang et al., 2018). The combination of its biological characteristics—cold resistance, asexual reproduction, and broad host specificity—facilitates the rapid establishment of populations and supports the enzootic maintenance of pathogen transmission cycles in newly colonized regions, thus posing significant threats to public health and veterinary disease management (Yu et al., 2023).

Among *Babesia* species*, B. microti* is the most prevalent zoonotic pathogen. Although approximately 2,000 annual cases are reported, epidemiological evidence indicates the true incidence is significantly higher (Bloch et al., 2019; Diuk-Wasser et al., 2014; Krause et al., 2003). Although *Ixodes* spp. are recognized as the primary vectors of*B. microti*, recent epidemiological surveillance has detected *B. microti* DNA in *H. longicornis*, suggesting a possible vectorial role (Zhang et al., 2017). Experimental transmission models have demonstrated that *H. longicornis* can acquire *B. microti* from infected murine hosts during blood feeding and subsequently transmit the parasite to naïve mice, thereby establishing its competence as an alternative transmission vector (Wu et al., 2017). Upon infection, *H. longicornis* initiates complex innate immune responses mediated by several effector molecules, including antimicrobial peptides (such as defensin, microplusin, and hebraein), protease regulators (Kunitz domain-containing proteins), transport molecules (lipocalins), and enzymatic regulators (proteases) (Antunes et al., 2017; De la Fuente et al., 2017; Wikel, 1999). However, these responses are often countered by the parasite's ability to exploit host-derived factors, thereby enhancing its colonization and transmission efficiency. While these vector-parasite interactions have been partially elucidated in other *Babesia*-tick systems, the precise molecular mechanisms underlying *B. microti* infection in *H. longicornis* remain poorly understood.

Unlike vertebrates, ticks lack adaptive immunity and rely solely on their innate immune mechanisms for defense against pathogens (Brossard and Wikel, 2004; Hart and Thangamani, 2021). This defense system comprises various immune cells and signaling molecules capable of pathogen recognition and elimination (Fogaça et al., 2021; Yuan et al., 2020). Programmed Cell Death (PCD), including apoptosis, autophagy, and ferroptosis, is a fundamental component of innate immunity and plays a crucial role in cellular homeostasis and developmental processes in eukaryotes (Jorgensen et al., 2017; Nagata and Tanaka, 2017). Notably, several tick-borne pathogens have evolved mechanisms to modulate host PCD pathways to facilitate their survival and transmission (Chen et al., 2021). For instance, *Rickettsia rickettsii* inhibits apoptosis in infected tick cells by suppressing *caspase-3* activity, thereby enhancing the growth and proliferation of the bacteria within the host cells (Martins et al., 2020). Similarly, *A. phagocytophilum* promotes intracellular survival by downregulating Porin expression, which decreases mitochondrial cytochrome C release and impairs apoptosis (Ayllón et al., 2015). Although bacterial modulation of autophagy pathways has been extensively studied in mammalian hosts infected with *Anaplasmataceae*, the role of autophagy in tick-pathogen interactions remains elusive (Lin et al., 2016; Niu et al., 2012). Our previous study demonstrated that *B. microti* infection upregulates the expression of Hemolymph-Related Factor (HRF) in the midgut of *H. longicornis*, inducing ferroptosis and promoting parasite colonization (Chen et al., 2025). However, the involvement of apoptosis and autophagy in tick responses to *B. microti* infection has not been fully elucidated, warranting further investigations.

In this study, a *B. microti*—mouse—*H. longicornis* infection model was established to investigate early-stage molecular interactions between *B. microti* and the midguts of *H. longicornis*. Dissected midgut tissues from engorged *H. longicornis* nymphs were subjected to RNA Sequencing (RNA-Seq) to assess transcriptomic changes associated with *B. microti* infection. Comparative analysis of infected and uninfected ticks identified Differentially Expressed Genes (DEGs) associated with apoptosis and autophagy. Functional validation using RNA interference (RNAi) demonstrated that silencing of *caspase-7, caspase-9*, and *ATG5* significantly decreased *B. microti* burden, indicating the parasite's dependence on these host cellular pathways for successful colonization. Furthermore, pharmacological modulation of autophagy with rapamycin (an autophagy activator) and 3-methyladenine (an autophagy inhibitor) demonstrated that *B. microti* modulates host cell PCD mechanisms to promote its survival. These findings provide novel insights into *Babesia*-ticks interactions and highlight potential molecular targets for transmission-blocking interventions against tick-borne babesiosis.

## 2 Materials and methods

### 2.1 Ethics statement

All experimental protocols were approved by the Institutional Animal Care and Use Committee and the Animal Ethics Committee of the Shanghai Veterinary Research Institute (Approval Nos. SHVRI-SZ-202008026-01, SHVRI-SV-20230616-03, and SHVRI-20230602-01).

### 2.2 *Babesia*, tick, and animal models

*B. microti* strains (ATCC PRA-99™; Manassas, VA, U.S.A.) were maintained in the laboratory through serial intraperitoneal passages in BALB/c mice. Female BALB/c mice (5–6 weeks old, 18–20 g) were obtained from Suzhou Sibifu Biotechnology Co., Ltd. (Suzhou, China) for parasite propagation and tick infection studies. Laboratory colonies of *H. longicornis* were maintained under controlled environmental conditions (25 °C, 60% relative humidity, complete darkness) and fed on New Zealand White rabbits supplied by the Shanghai Laboratory Animal Center (Chinese Academy of Sciences).

### 2.3 microti infection in H. longicornis

Tick infection with *B. microti* was conducted following previously established protocols (Wu et al., 2017). Cryopreserved *B. microti* strains (ATCC PRA-99™) were rapidly thawed in a 37 °C water bath, and 500 μL of the suspension was administered intraperitoneally into specific pathogen-free BALB/c mice. *B. microti* was monitored daily through microscopic examination of thin peripheral blood smears stained with 10% Giemsa solution (pH 7.2). *B. microti* infection was typically confirmed within 5-−7 d post-inoculation. Upon reaching a *B. microti* level of 50%, blood was collected into EDTA-coated tubes, and 200 μL aliquots were used to infect naïve, age-matched immunocompetent mice to maintain the infection cycle. For tick exposure, 60 *H. longicornis* nymphs were applied to the shaved dorsal skin of each *B. microti*-infected mouse (10−15%) and allowed to feed to repletion. This method was used for all groups. This time point was selected to synchronize the rapid engorgement phase of ticks with peak *B. microti*, thereby optimizing the efficiency of pathogen acquisition.

## 3 Quantitative detection of *B. microti*

Quantification of *B. microti* burden was performed using a TaqMan probe-based quantitative PCR (qPCR) assay following established protocols (Persing et al., 1992; Rollend et al., 2013). A 429-bp fragment of the *B. microti* 18S rDNA (GenBank accession no. AB190435.1) was cloned into a pMD18-T vector (TaKaRa Bio, Japan) to generate a standard curve using serial ten-fold dilutions (10^1^ – 108 copies/μL). qPCR analysis was conducted in triplicate on a QuantStudio 5 Real-Time PCR System (Applied Biosystems, U.S.A.). Each 20 μL reaction contained 10 μL 2 × Premix Ex Taq (Hot Start DNA polymerase), 0.6 μL of each primer (10 μM), 0.3 μL of FAM/BHQ1-labeled probe (10 μM), and 3 μL of DNA template. The thermal cycling conditions included an initial denaturation at 95°C for 30 s, followed by 40 amplification cycles of 95°C for 5 s and 60°C for 34 s. Fluorescence signals were recorded at the end of each extension phase. Primer and probe sequences are provided in Supplementary Table 1. Each qPCR run included negative controls (no template) and inter-run calibrators to ensure the specificity, sensitivity, and reproducibility of the assay.

### 3.1 Midgut collection from *H. longicornis*

Engorged *H. longicornis* nymphs were collected from both *B. microti*-infected and uninfected groups at two critical time points: 0 day post-engorgement and 4 d post-engorgement. Each biological replicate consisted of a pooled sample of thirty ticks, with three replicates per group (*n* = 3). Ticks were surface-sterilized by immersion in 70% ethanol with gentle agitation (100 rpm) for 90 s, followed by three sequential washes in sterile phosphate-buffered saline (PBS, pH 7.4) to remove residual ethanol. Following meticulous dissection with fine forceps to isolate midguts and prevent contamination from neighboring tissues (e.g., salivary glands and reproductive organs), samples were immediately transferred to pre-cooled PBS. Each midgut was then incised using sterile surgical blades and subjected to three sequential washes with PBS to ensure the complete removal of residual hemolymph components. Finally, samples were promptly flash-frozen in 500 μL of RNAlater Stabilization Solution (Thermo Fisher Scientific) to preserve RNA integrity.

### 3.2 RNA extraction and qPCR

Total RNA was extracted from the dissected midgut tissues using TRIzol reagent (Invitrogen), according to the manufacturer's protocol. Frozen samples were thawed on ice and homogenized in 1 mL TRIzol reagent per 50-−100 mg of tissue. Following a 5 min incubation at room temperature, 0.2 mL chloroform was added per 1 mL of TRIzol, and the mixture was vigorously shaken for 15 s before centrifugation at 12,000 × *g* for 15 min at 4 °C. The aqueous phase, containing the RNA, was carefully transferred to a new RNase-free tube and mixed with an equal volume of isopropanol to precipitate RNA. After centrifugation at 12,000 × *g* for 10 min at 4 °C, the supernatant was discarded, and the resulting RNA pellet was washed twice with 75% ethanol, air-dried, and resuspended in RNase-free water. RNA concentration and purity were assessed using a NanoDrop spectrophotometer (Thermo Scientific), and the integrity was verified by agarose gel electrophoresis, ensuring a RIN > 8.0. RNA Aliquots were stored at −80 °C until further use. All procedures were performed under RNase-free conditions using DEPC-treated materials to minimize RNA degradation.

The RNA was converted to first-strand cDNA using a HiScript III RT SuperMix for qPCR (gDNA wiper) kit (Vazyme Biotech, China). The cDNA was used to analyze the relative quantitative changes in gene expression (Supplementary Table 2). Samples were subjected to qRT-PCR using ChamQ Universal SYBR qPCR Master Mix (Q711, Vazyme) in a QuantStudio™5 Real-Time PCR System (Applied Biosystems™, New York, U.S.A.), and all samples were analyzed with three replicates. Elongation factor-1 (ELF1A, GenBank registry number AB836665) is an internal control for relative gene expression (following the 2^−Δ*ΔCt*^ method) (Nijhof et al., 2009).

### 3.3 RNA-seq and transcriptomic analysis

High-quality total RNA samples were submitted to Omicsmart (China) for transcriptomic sequencing. Ribosomal RNA (rRNA) was depleted from the RNA samples using the Ribo-Zero Globin kit (Illumina, San Diego, CA, USA), and the enriched mRNA was fragmented and reverse-transcribed into first-strand cDNA using random hexamer primers. Second-strand cDNA synthesis was performed using a reaction mixture containing buffer, dNTPs (substituting dUTP for dTTP), RNase H, and DNA polymerase I. The resulting double-stranded cDNA was purified using a QiaQuick PCR purification kit (Qiagen) and subjected to end repair, adenine (A)-tailing, and adapter ligation to generate sequencing libraries. Second-strand cDNA was selectively degraded using Uracil-N-Glycosylase (UNG) to ensure strand specificity during sequencing. Library fragments were size-selected using agarose gel electrophoresis and amplified by PCR.

Sequencing was performed on the Illumina HiSeqTM 4000 platform, generating 150 bp paired-end reads. Raw sequencing reads were quality-filtered to obtain clean reads, which were subsequently aligned to the reference genome of *H. longicornis* using HISAT2 v2.1.0 (http://daehwankimlab.github.io/hisat2/). Transcript assembly and quantification were performed using StringTie v1.3.4 (https://ccb.jhu.edu/software/stringtie/index.shtml), enabling the identification of both annotated and novel transcripts. Gene expression levels were quantified across all samples based on the HISAT2 alignments. Differential gene expression analysis was conducted using the edgeR 3.12.1 (http://www.bioconductor.org/packages/release/bioc/html/edgeR.html). Read counts were normalized, and statistical significance was evaluated using negative binomial models, with False Discovery Rate (FDR) correction for multiple comparisons. Differentially Expressed Genes (DEGs) were defined based on the thresholds of FDR < 0.05 and |log2FC| > 1. Functional enrichment analyses were performed by mapping identified DEGs to the Gene Ontology (GO) (https://www.bioconductor.org/packages/release/data/annotation/html/GO.db.html) and Kyoto Encyclopedia of Genes and Genomes (KEGG) databases (http://www.kegg.jp). Significantly enriched GO terms and KEGG pathways (*P* < 0.05) were identified, providing mechanistic insights into the transcriptomic responses of *H. longicornis* midgut tissue to *B. microti* infection and highlighting key host-pathogen interactions.

### 3.4 TUNEL assay

Midguts from engorged *H. longicornis* nymphs were dissected in PBS, fixed in 4% paraformaldehyde (PFA) at 4 °C for 24 h, dehydrated, and embedded in paraffin, and sectioned at a thickness of 5 μm. Tissue sections were deparaffinized, rehydrated, and subjected to proteinase K digestion (20 μg/mL, 37 °C, 30 min) to facilitate antigen retrieval, followed by permeabilization with 0.1% Triton X-100 on ice for 10 min. TUNEL staining was performed using a commercially available kit (Roche) following the manufacturer's instructions. Sections were incubated with a terminal deoxynucleotidyl transferase (TdT)/FITC-dUTP labeling mixture at 37 °C for 1 h. Negative (TdT) and positive (+DNase I) controls were included to confirm assay specificity. Nuclei were counterstained with DAPI and visualized using a fluorescence microscope equipped with appropriate filter sets. Apoptotic rates were calculated by TUNEL?/DAPI? percentages across three randomly selected fields.

### 3.5 Transmission electron microscopy (TEM)

This study used the same *H. longicornis* midgut epithelial cell samples and experimental methods as in our previous publication to observe the autophagosome (Magnification,7,000 × ) following *B. microti* infection (Chen et al., 2025). Specifically, dissected midguts were washed thrice with Phosphate-Buffered Saline (PBS) and fixed overnight at 4 °C in 2.5% glutaraldehyde. Subsequent post-fixation was performed using 1% osmium tetroxide (OsO4) in 0.1 M phosphate buffer (PBS; pH 7.4) for 2 h in the dark. After three PBS rinses (15 min), samples were dehydrated in a graded ethanol series (30−100%, 20 min), acetone-embedded, and polymerized (37°C overnight). Ultrathin sections (60-−80 nm) were mounted on copper grids and double-stained with 2% uranyl acetate and 2.6% lead citrate for 8 min in a CO_2_-free environment. Sections were air-dried and imaged using a HITACHI transmission electron microscope.

### 3.6 RNAi

Ticks were treated with gene-RNA interference (RNAi) according to previously published methods using the primers listed in Supplementary Table 3. Gene-specific RNA interference primers were designed against *caspase-7, caspase-9*, and ATG5 sequences from our transcriptome database, each incorporating a5′-T7 RNA polymerase promoter sequence, with *Luciferase* serving as the normalization control. Double-stranded RNA (dsRNA) was synthesized using the T7 RiboMAX™ Express RNAi System (Promega, Madison, WI, U.S.A.) following the manufacturer's protocol. Briefly, target-specific DNA fragments (200−500 bp) flanked by T7 promoters were transcribed at 37°C for 4 h, followed by thermal denaturation and controlled annealing to generate dsRNA. The product was treated with DNase I (15 min, 37°C) to eliminate template DNA, purified by ethanol precipitation, and quantified spectrophotometrically. For tick RNAi, 23 nL (10 μg/μL) of synthesized dsRNA was precisely injected into the root of the last pair of legs of the nymphs using a microinjector (Drummond Scientific, U.S.A.). Interference-treated ticks were left to stand for 12 h (*n* = 50) and then fed simultaneously with controls (*n* = 50) on the same mice infected with *B. microti*. Two engorged nymphs were assigned to each group, with at least five biological replicates included, followed by DNA extracted from the ticks for *B. microti* detection. One limitation of this study is that the susceptibility of each mouse to *B. microti* varies, which resulted in inter-batch and inter-group variations.

### 3.7 Rapamycin and 3-methyladenine treatment

The effects of autophagy on *B. microti* infection were investigated using the autophagy inducer Rapamycin (Beyotime # S1842) and the inhibitor 3-methyladenine (Solarbio #IM0190). Those were microinjected into engorged nymphs infected with *B. microti* at a volume of 69 *n*L (Feng et al., 2021). DMSO was used as a control, and samples were collected 3 d after injection.

### 3.8 Data analysis

Statistical analyses were performed using GraphPad Prism 6 software (GraphPad Software Inc., San Diego, CA, U.S.A.). Quantitative data were expressed as mean ± Standard Deviation (SD). Intergroup comparisons were conducted using the two-tailed Mann–Whitney U test, unpaired Student's *t* test, or one-way analysis of variance (ANOVA), as appropriate. A *P* value < 0.05 was considered statistically significant.

## 4 Results

### 4.1 RNA-seq of *B. microti*-infected ticks

**Figure 1:**
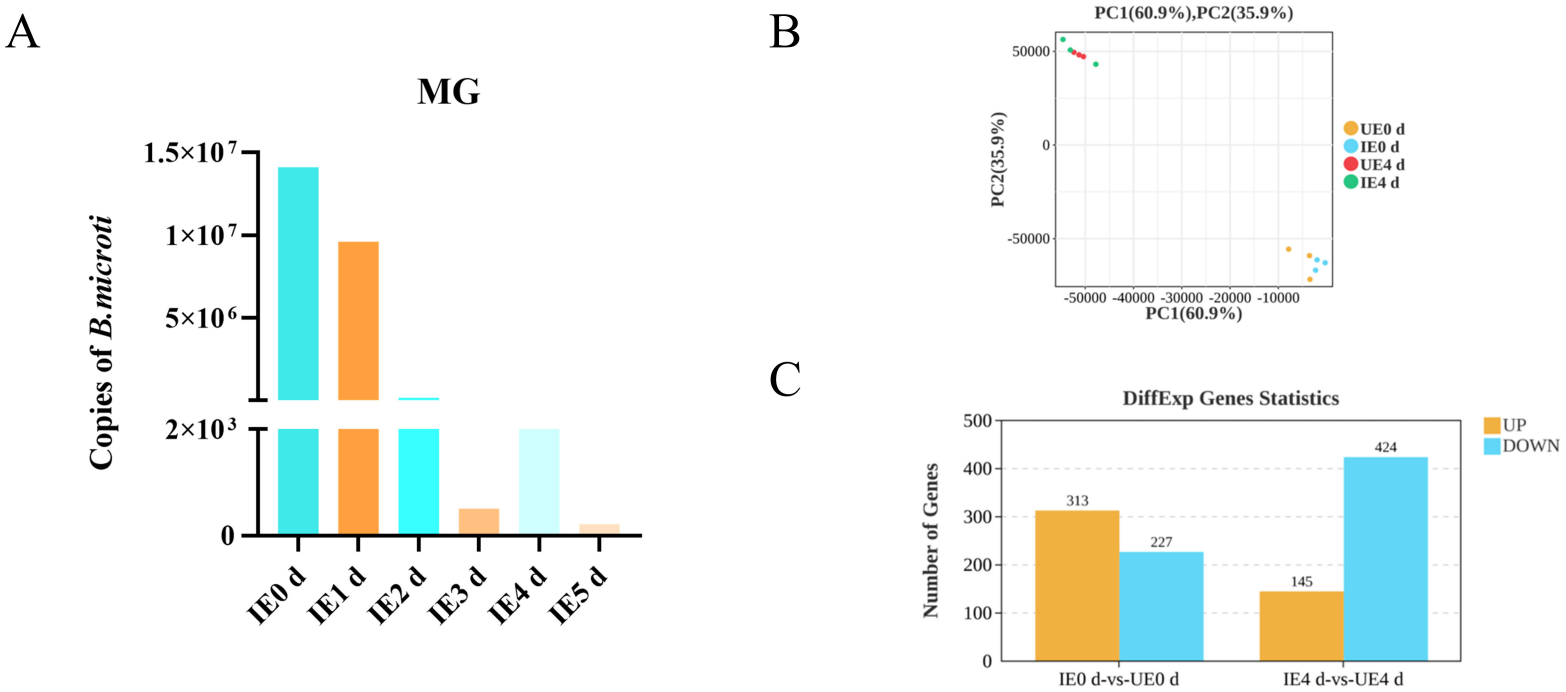
*B. microti* presence and differential gene expression in *B. microti*-infected tick midguts. **(A)** *B. microti* presence in the engorged nymph midgut (*n* = 3). **(B)** Principal component analysis (PCA) of gene expression profiles. **(C)** Upregulated and downregulated genes are indicated in red and blue, respectively. IE0 *d*: Infected *B. microti* at 0 day post-engorgement; IE1 *d*: Infected *B. microti* at 1 d post-engorgement; IE4 *d*: Uninfected *B. microti* at 4 d post-engorgement; UE0 *d*: Uninfected *B. microti* at 0 day post-engorgement; UE4 d: Uninfected *B. microti* at 4 d post-engorgement.

*B. microti* invade the tick midgut, differentiate into gametes, form a syncytium that migrates to the hemolymph and salivary glands (Jalovecka et al., 2019). We quantified *B. m*icroti in the midguts of engorged nymphal ticks by qPCR (*n* = 10 per group, with three biological replicates). The analysis demonstrated that parasite load peaked immediately after engorgement. Subsequently, a significant decline in parasite numbers was observed between d 1 and 3 post-engorgement. A transient rebound in parasite load occurred on day 4, followed by a further reduction on d 5 and 6 (Figure 1A). Principal Component Analysis (PCA) was conducted using the gmodels package in R to assess the variance in gene expression data. The resulting PCA plot revealed a strong tendency for biological replicates within each experimental group to cluster tightly, suggesting a high degree of reproducibility and reliability in the experimental data (Figure 1B). RNA-seq was performed on midgut tissues from *H. longicornis* nymphs at 0 and 4 d post-engorgement following *B. microti* infection based on quantitative detection of *B. microti* in engorged nymphal midguts (*n* = 10, 3 replicates). Raw sequencing reads from infected and uninfected midgut underwent stringent quality assessment before bioinformatic analysis at 0 and 4 d post-engorgement. Each sample generated over 4 GB of clean reads, with >99% read retention. The GC content ranged between 48% and 50%, and Q30 scores consistently exceeded 95%, meeting established quality thresholds for transcriptomic analyses. Clean reads were aligned to the *H. longicornis* reference genome (ASM966319v1) using HISAT2, achieving alignment rates greater than 50% across all samples. While this moderate mapping efficiency likely reflects genomic divergence between the reference bisexual strain and the parthenogenetic colony used in this study, the high Q30 scores (>93%) and stable GC content confirm the reliability of the dataset for downstream transcriptomic profiling (Table 1).

**Table 1:** Overview of RNA sequencing data.

**Sample**	**Raw reads**	**Clean reads (%)**	***Q*30**	**GC content**
UE0 *d*1	37554142	37530376 (99.94%)	95.47%	49.57%
UE0 *d*2	49747146	49706630 (99.92%)	96.44%	48.80%
UE0 *d*3	43351384	43301264 (99.88%)	96.54%	49.05%
IE0 0*d*1	43007750	42962060 (99.89%)	96.45%	49.57%
IE0 0*d*2	47082488	47039006 (99.91%)	96.59%	47.64%
IE0 0*d*3	49790492	49745054 (99.91%)	96.44%	47.30%

Comparative transcriptomic analysis revealed significant temporal changes in midgut gene expression in response to *B. microti* infection. At 0 d post-engorgement, 540 DEGs were identified, comprising three hundred and thirteen upregulated and two hundred and twenty seven downregulated transcripts (FDR < 0.05). By 4 d post-engorgement, 569 DEGs were identified, including one hundres and forty five upregulated and four hundred and twenty four downregulated, indicating a shift toward global transcriptional suppression (Figure 1C, Supplementary Tables 4, 5).

### 4.2 GO annotation

**Figure 2:**
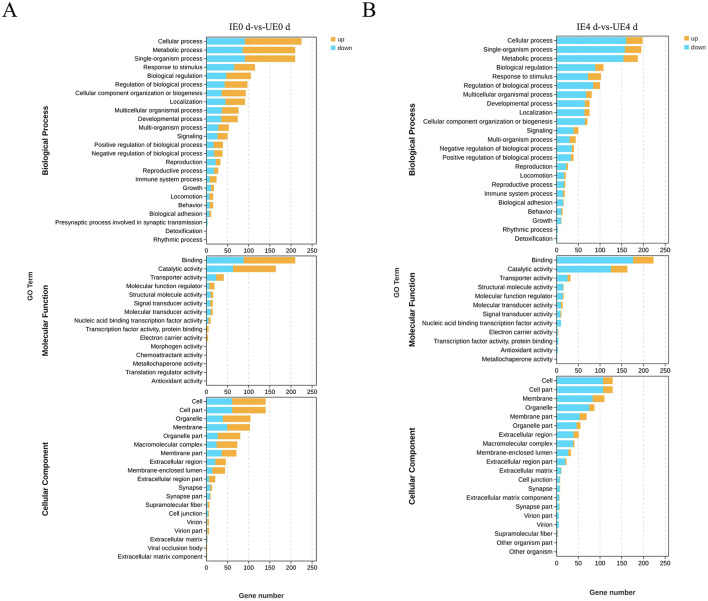
GO enrichment analysis of DEGs in tick midguts at 0 **(A)** and 4 days **(B)** post-engorgement. Bar plots show significantly enriched GO terms across three functional categories: Biological Process (BP); Molecular Function (MF); and Cellular Component (CC). The x-axis represents the number of DEGs associated with each GO term, while the y-axis indicates the corresponding GO terms. Upregulated terms are shown in yellow, while downregulated terms are in blue. IE0 *d*: Infected *B. microti* at 0 day post-engorgement; IE4 *d*: Infected *B. microti* at 4 d post-engorgement; UE0 *d*: Uninfected *B. microti* at 0 day post-engorgement; UE4 *d*: Uninfected *B. microti* at 4 d post-engorgement.

GO enrichment analysis of DEGs revealed temporally distinct functional responses in the midgut during *B. microti* infection. At 0 d post-engorgement, fifty eight significantly enriched GO terms (FDR < 0.05) were identified, comprising twenty four biological processes, fifteen molecular functions, and nineteen cellular components (Figure 2A). At 4 d post-engorgement, fifty five significantly enriched GO terms were identified, including twenty three biological processes, twelve molecular functions, and twenty cellular components, indicating persistent but restructured transcriptional activity (Figure 2B). Enriched biological processes included key cellular and metabolic processes, responses to external stimuli, developmental regulation, and cellular localization. Molecular functions were significantly associated with protein binding domains (particularly receptor-ligand interactions), enzymatic activity, and membrane transport. Enriched cellular components were related to plasma membrane structures, supramolecular complexes, and organelle luminal compartments (Figure 2).

### 4.3 KEGG pathway enrichment analysis

KEGG pathway analysis of DEGs revealed significant enrichment in six functional categories in response to *B. microti* infection: human disease, organismal systems, metabolism, genetic information processing, and cellular processes.

At 0 d post-engorgement, *B. microti* infection significantly perturbed several key biological pathways in *H. longicornis* (Supplementary Figure 1A). Pathway enrichment analysis identified significant alterations (Q < 0.05) in immune system processes, including antigen processing and presentation; metabolic pathways, such as steroid hormone biosynthesis, and linoleic acid metabolism; and digestive system functions, including protein digestion and absorption, pancreatic juice secretion, and mineral absorption. Furthermore, pathways associated with specific diseases, including Legionellosis, Toxoplasmosis, and Measles, were also significantly enriched. Although not reaching statistical significance (Q > 0.05), the cellular processes of apoptosis and autophagy exhibited a trend toward enrichment. These findings collectively suggest that *B. microti* infection elicits broad effects on a range of physiological functions in the tick host, encompassing immune responses, metabolic regulation, and nutrient absorption, even at the early stages of infection.

Although pathway enrichment analysis at 4 d post-engorgement did not identify statistically significant results (Q > 0.05), examining the top thirty pathways demonstrating trend changes revealed potentially relevant regulatory shifts (Supplementary Figure 1B). These pathways encompass processes related to the immune system, such as complement and coagulation cascades; the digestive system, including protein digestion and absorption; and cellular processes, including lysosome, apoptosis, and autophagy. While these pathways did not meet the threshold for statistical significance, they warrant further investigation as potential targets of regulation following engorgement.

### 4.4 Validation of RNA-seq findings by qRT-PCR

**Figure 3:**
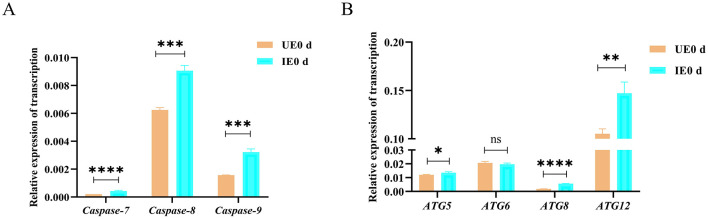
Transcriptional activation of apoptosis and autophagy pathways in tick midguts following *B. microti* infection **(A)** qRT-PCR analysis of apoptosis-related genes (*caspase-7, caspase-8*, and *caspase-9*) and in *H. longicornis* nymphs at day 0 post-engorgement (*n* = 3). **(B)** autophagy-related genes (*ATG5, ATG6, ATG8, ATG12*) in *H. longicornis* nymphs at day 0 post-engorgement (*n* = 3). *B. microti*-infected midguts (green) exhibited significant upregulation of apoptosis- and autophagy-related genes compared with uninfected controls (red). Data are presented as the mean ± standard error. *P* < *0.05;* **P < *0.01;* ***P < *0.001;* ****P < *0.0001*, differential gene expression analysis determined using Student's *t* test.

Given the important role of cellular processes in host-pathogen interactions, pathway enrichment analysis suggests that apoptosis and autophagy pathways may be involved in *B. microti* infection processes (Chen et al., 2021). To validate the transcriptomic results, the expression levels of key apoptosis-related genes (*caspase-7, caspase-8*, and *caspase-9*) and autophagy-related genes (*ATG5, ATG6, ATG8*, and *ATG12*) were analyzed by qRT-PCR at 0 d post-engorgement. These genes were selected based on their significant upregulation (*P* < 0.05) in the RNA-seq dataset and their established roles in cellular stress response, particularly apoptosis and autophagy. Our analysis revealed significant upregulation of key apoptosis-related genes, including *caspase-7* (*P* = *0.022*), *caspase-8* (*P* = *0.0003*), and *caspase-9* (*P* = *0.0003*), in response to infection (Figure 3A). Similarly, autophagy-related genes *ATG5* (*P* = *0.029*), ATG8 (*P* < *0.0001*), and ATG12 (*P* = 0.0045) showed marked transcriptional activation, while ATG6 (*P* = *0.28*) and expression remained unchanged (Figure 3B). The qRT-PCR results corroborated the RNA-seq findings, demonstrating consistent and statistically significant upregulation of most selected transcripts. These results validated the reliability of the RNA-seq data, highlighting the activation of apoptotic and autophagic pathways in tick midgut following *B. microti* infection.

### 4.5 *B. microti* infection induces autophagy and apoptosis in tick midgut

**Figure 4:**
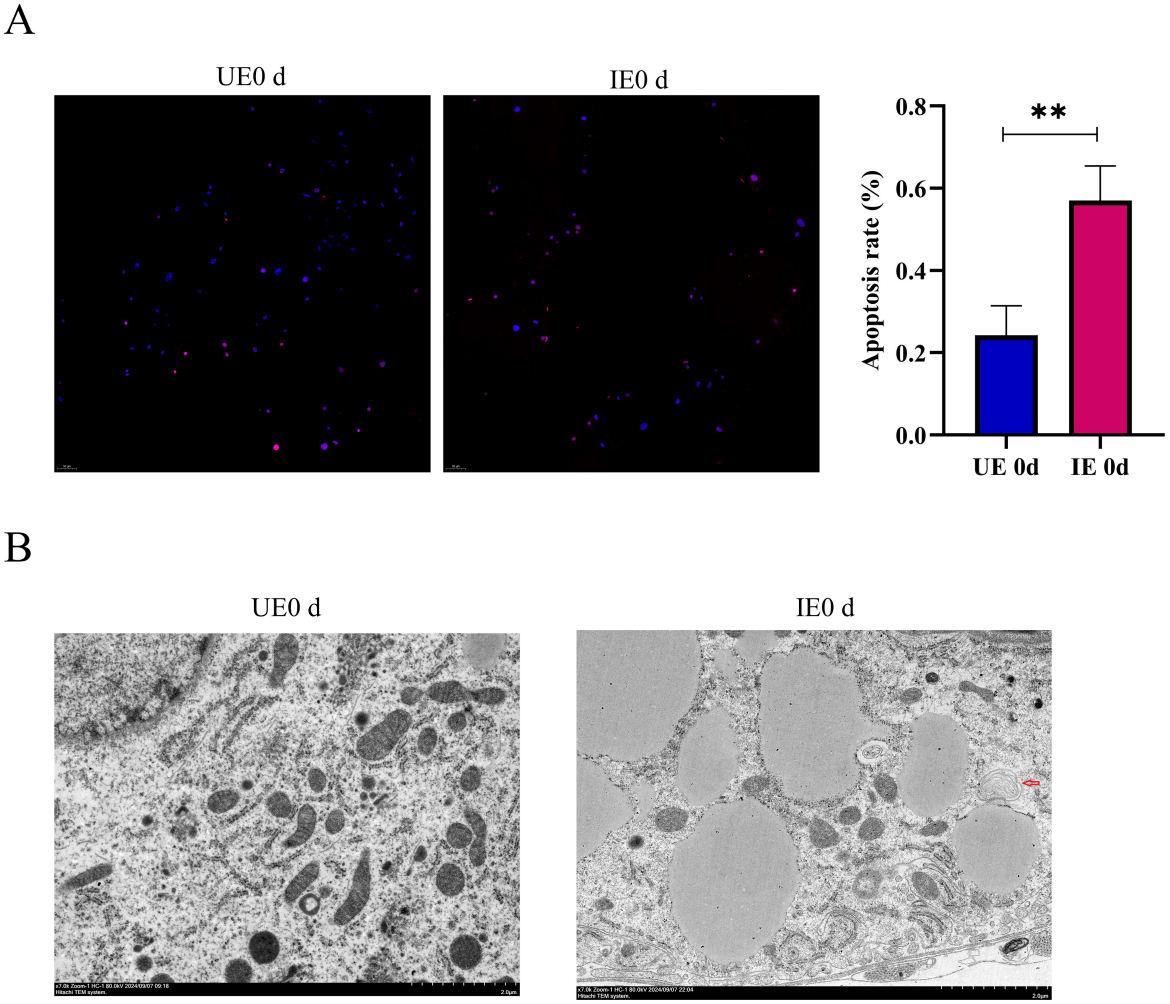
*B. microti* infection induces apoptosis and autophagy in tick midguts. **(A)** Apoptosis assessment in midgut tissues of engorged ticks by TUNEL staining (*n* = 3). Nuclei were counterstained with DAPI (blue) and apoptotic cells were labeled with TUNEL (red). Quantitative analysis of fluorescence intensity **(right)** showed significantly elevated apoptosis levels in *B. microti*-infected ticks. **(B)** TEM revealed autophagy activation in *B. microti*-infected ticks, indicated by characteristic double- or multi-membrane vesicles enclosing cytoplasmic contents (arrows, *n* = 3). This experiment was conducted concurrently with the mitochondrial observations reported by our previous publication, utilizing the same biological samples for autophagosome observation (Chen et al., 2025). UE0 *d:* Uninfected *B. microti* at 0 day post-engorgement; IE0 *d*: infected *B. microti* at 0 day post-engorgement. Data are presented as the mean ± standard error. *P* < *0.05;* **P < *0.01;* ***P < *0.001;* ****P < *0.0001*. differential gene expression analysis determined using Student's *t* test.

Apoptosis and autophagy are tightly regulated cellular processes essential for maintaining tissue homeostasis and modulating host responses to pathogen invasion. Apoptosis is characterized by distinct nuclear morphological changes, including chromatin condensation, nuclear fragmentation, and karyolysis (Jiang et al., 2000). In contrast, autophagy involves the sequestration of damaged organelles or misfolded proteins within double-membrane autophagosomes, which subsequently fuse with lysosomes for degradation. To evaluate the impact of *B. microti* infection on apoptosis, TUNEL staining was performed on the tick midgut at 0 d post-engorgement. The results revealed a significant increase in fluorescence signal intensity in the midgut tissues of the infected group compared with the control (*P* < 0.05), indicating elevated apoptotic activity (Figure 4A). Complementary ultrastructural analysis by TEM revealed characteristic autophagic structures, including double- and multi-membrane-bound vesicles, within the midgut epithelial cells of infected ticks at 0 day post-engorgement, suggesting enhanced autophagic activity during early infection (Figure 4B, Supplementary Figure 2). These findings collectively indicate that *B. microti* infection concurrently activates apoptotic and autophagic pathways in tick midgut cells, highlighting their potential synergistic role in host-pathogen interactions.

### 4.6 *Caspase-7 and caspase-9* regulate tick acquisition of *B. microti*

**Figure 5:**
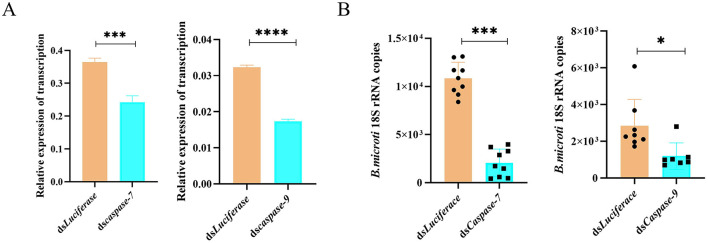
RNAi of *caspase-7* and *caspase-9* reduces *B. microti* infection in *H. longicornis*. **(A)** qPCR analysis of RNAi efficiency for *caspase-7* and *caspase-9* in engorged nymphs (*n* = 3). **(B)** qRT-PCR analysis demonstrated a significant reduction in *B. microti* load following *caspase-7* (*n* = 9) *and caspase-9* (*n* = 6) gene silencing in *H. longicornis* nymphs compared with *luciferase* dsRNA controls (*n* = 9 for *caspase-7* comparison; *n* = 8 for *caspase-9* comparison). Data are presented as the mean ± standard error. *P < *0.05;* **P < *0.01;* ***P < *0.001;* ****P < *0.0001*, differential gene expression analysis determined using Student's *t* test; *B. microti* load analysis determined using two-tailed Mann-Whitney U test.

To evaluate the functional role of apoptosis in *B. microti* acquisition, RNAi was used to silence the apoptotic regulators *caspase-7* and *caspase-9* in *H. longicornis* nymphs. Caspase-7 functions as an executioner caspase mediating the terminal phase of apoptosis in mammalian systems, whereas *caspase-9* serves as an initiator *caspase* in the intrinsic (mitochondrial) apoptotic pathway (An et al., 2020; Ghazavi et al., 2022). Quantitative reverse transcription PCR (qRT-PCR) analysis confirmed the successful knockdown of *caspase-7* (*P* = *0.0007*) and *caspase-9* (*P* < *0.0001*) expression in nymphal ticks achieved through RNAi (Figure 5A). Gene knockdown of both *caspases* resulted in a significant reduction in *B. microti* load in infected ticks compared with the control at 0 d post-engorgement, indicating that these caspases are essential for efficient *B. microti* establishment in the ticks (Figure 5B). The observed variation in *18S rRNA* copy number among control samples reflects biological differences in parasite acquisition, rather than technical or sampling errors.

### 4.7 Autophagy enhances tick susceptibility to *B. microti* infection

**Figure 6:**
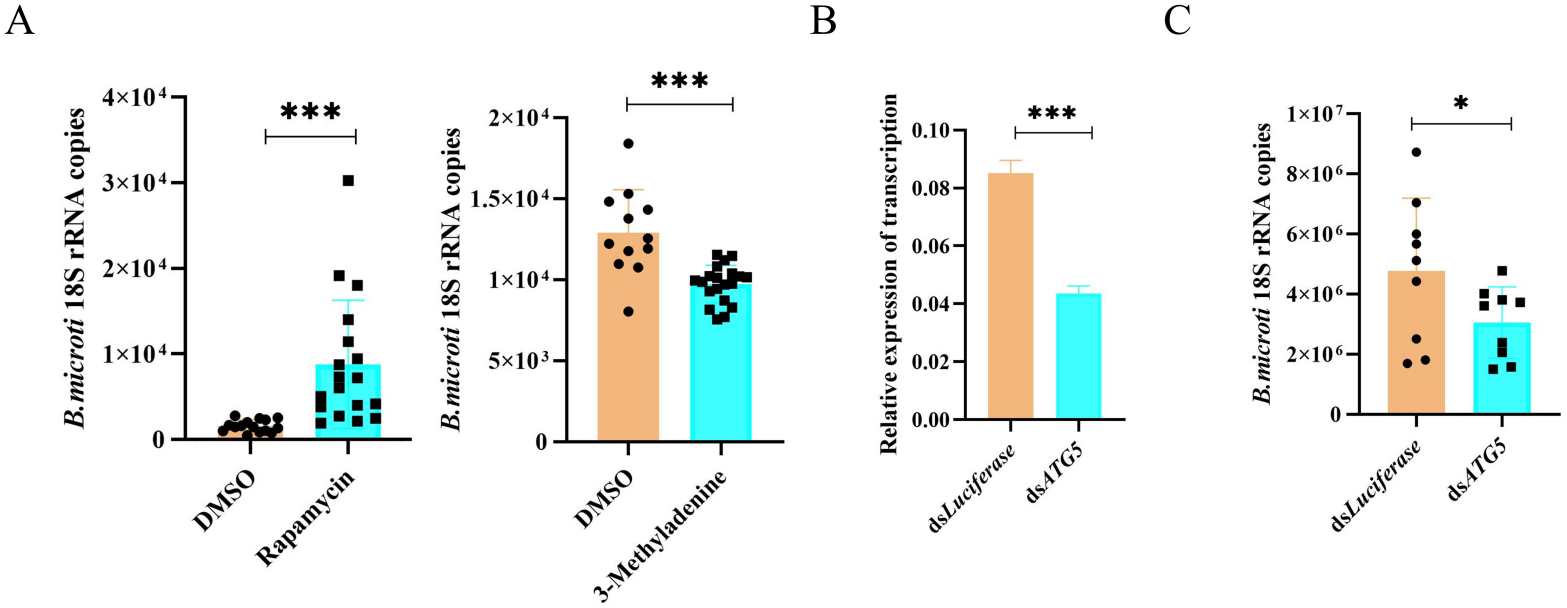
Autophagy promotes *B. microti* infection in *H. longicornis*. **(A)** Differential analysis of *B. microti* load in ticks treated with rapamycin (autophagy inducer, *n* = 18), 3-methyladenine (autophagy inhibitor, *n* = 20) and DMSO (*n* = 15 for rapamycin comparison; *n* = 12 for 3-methyladenine comparison). **(B)** qPCR analysis assessing RNAi efficiency for *ATG5* genes in engorged tick nymphs (*n* = 3). **(C)** qRT-PCR analysis revealed that *ATG5* knockdown significantly reduced *B. microti* acquisition in *H. longicornis* nymphs (*n* = 9). Data are presented as the mean ± standard error. *P < *0.05;* **P < *0.01;* ***P < *0.001;* ****P < *0.0001*, differential gene expression analysis determined using Student's *t* test; *B. microti* load analysis determined using two-tailed Mann-Whitney U test.

To further investigate the role of autophagy in *B. microti* infection, pharmacological modulation of autophagy was performed in infected ticks. Rapamycin (10 mM), an autophagy activator, and 3-MA (5 mM), an autophagy inhibitor, were microinjected into *B. microti*-infected ticks post-engorgement. Parasite burden was assessed 3 d post-treatment for each group, respectively. Rapamycin treatment significantly increased the *B. microti* load, whereas 3-MA significantly decreased the parasite burden, indicating that enhanced autophagic activity promotes *B. microti* survival (Figure 6A). To confirm the genetic basis of this observation, RNAi was conducted to silence *ATG5* (*P* = *0.0001*), a critical gene implicated in autophagosome formation and cross-regulation with apoptosis (Figure 6B). *ATG5* knockdown significantly reduced *B. microti* load in tick midgut tissues, corroborating the pharmacological results (Figure 6C). These findings suggest that autophagy facilitates *B. microti* infection in *H. longicornis*, enhancing tick susceptibility to the parasite. Therefore, targeting autophagy-related pathways may represent a novel strategy to reduce vector competence and limit transmission of tick-borne babesiosis.

## 5 Discussion

The transmission dynamics of *B. microti* primarily involve horizontal acquisition by tick larvae and nymphs during blood feeding, rather than transovarial transmission (Florin-Christensen et al., 2021; Wu et al., 2017). Consequently, the tick midgut and salivary glands serve as the principal sites for *B. microti* acquisition, replication, and transmission to vertebrate hosts (Gray et al., 2002; Rudzinska et al., 1983). Despite the crucial role of the midgut in vector competence, the specific molecular mechanisms underlying the complex interactions between *Babesia* spp. and their tick vectors in this tissue remain poorly understood. This study characterized tick midgut transcriptional responses to *B. microti* infection at 0 and 4 d post-engorgement. RNA-seq revealed significant autophagy and apoptosis pathway activation, validated by qRT-PCR and histology. The coordinated induction of these processes suggests *B. microti* manipulates host cell death pathways to enhance midgut infection, providing new insights into vector-pathogen adaptation.

Comparative transcriptomic analyses of *B*. microti-infected ticks during the early stages of infection revealed significant enrichment of metabolic and cellular processes, particularly those associated with nutrient transport, energy metabolism, cell proliferation, and cell death. Unlike previous transcriptomic studies that analyzed whole engorged nymphs, this study specifically focused on the tick midgut—the primary site for *B. microti* invasion and establishment, thus providing tissue-specific insights into tick-pathogen interactions at this critical interface (Feng et al., 2025). The whole transcriptome analysis identified a total of 1,051 DEGs, with a predominance of upregulated genes. Similarly, this study demonstrated a significant increase in upregulated genes following *B. microti* infection in the midgut of *H. longicornis*. This trend aligns with findings in other tick species, such as *Amblyomma aureolatum*, where *Rickettsia rickettsii* infection resulted in a significantly increased number of upregulated genes within the midgut compared with down regulated genes (Martins et al., 2017). The preferential upregulation of gene expression levels in response to *B. microti* infection suggests a selective activation of host immune and metabolic pathways rather than global suppression of host transcription. Given the midgut's critical role as the primary site for *B. microti* colonization and as the initial immunologic and physiologic barrier to pathogen invasion, the observed transcriptional changes likely reflect key host-pathogen interactions, particularly within pathways related to immunity, digestion, lipid metabolism, infectious disease, and cell growth (Cabezas-Cruz et al., 2019; De la Fuente et al., 2017). The predominance of upregulated genes in the midgut transcriptome indicates the tick's active counter-response aimed at limiting parasite proliferation through mechanisms such as antimicrobial peptide synthesis, oxidative stress induction, or metabolic reprogramming—a hypothesis requiring further functional validation.

Apoptosis and autophagy emerged as the predominant cell death pathways significantly enriched among upregulated Coding Sequences (CDSs) in the *B. microti-*infected *H. longicornis* nymphal midgut, highlighting their critical roles in modulating tick-pathogen interactions. Apoptosis, a highly regulated form of PCD, has been increasingly recognized for its role in modulating tick-pathogen interactions. Several studies have explored the potential mechanisms underlying apoptosis in tick cells and how pathogens manipulate these processes to enhance their survival and replication within the tick vector (Wang and Cull, 2022). While typically activated as a defense mechanism in response to cellular damage or infection, several tick-borne pathogens have evolved strategies to modulate apoptotic signaling to enhance their survival. For instance, *Anaplasma phagocytophilum* infection in *I. scapularis* induces host cell apoptosis, which paradoxically restricts bacterial proliferation by eliminating infected cells (Ayllón et al., 2015). However, under certain conditions, apoptosis may facilitate pathogen invasion and colonization. Specifically, the apoptosis-associated protein porin in ticks has been shown to promote *Babesia* infection during the early stages of tick blood engorgement (Zheng et al., 2020). These context-dependent effects of apoptosis suggest that, in some cases, this pathway may support pathogen infection and transmission. The present study supports this hypothesis, demonstrating increased apoptotic activity in tick midgut tissues following *B. microti* infection, ultimately enhancing parasite burden, as evidenced by RNAi-mediated silencing of *caspase-7, caspase-9*. Notably, *Babesia* infection exhibits tissue-specific modulation of host cell death pathways in ticks. Transcriptomic analyses of *B. bovis* and *B. bigemina-*infected *Rhipicephalus microplus* hemolymph reveal significant suppression of apoptotic pathways (Vimonish et al., 2025). This study provides the first experimental evidence that the apoptotic machinery of *H. longicornis* contributes to *B. microti* acquisition, highlighting *caspase-7* and *caspase-9* as potential molecular targets for transmission-blocking intervention against tick-borne babesiosis.

Autophagy, a conserved cellular degradation process, mediates lysosomal degradation of damaged organelles, misfolded proteins, and intracellular pathogens through the formation of double-membrane autophagosomes (Siqueira et al., 2018). Although autophagy typically functions as a host defense mechanism, certain tick-borne pathogens can exploit this process to enhance their intracellular survival. For instance, *A. phagocytophilum* secretes the effector protein Ats-1, which interacts with Beclin-1 to induce autophagosome formation, creating a nutrient-rich niche favorable to pathogen replication (Niu et al., 2012). Similarly, *Rickettsia buchneri* activates autophagy in tick cells to enhance its proliferation (Wang et al., 2024). In contrast, *Plasmodium vivax* infections induce autophagy in *Anopheles aquasalis* mosquitoes, leading to a reduction in parasite load and transmission potential (Santana et al., 2019). RNAi-mediated knockdown of the autophagy gene *ATG5* significantly reduced parasite burden, whereas pharmacological induction of autophagy promoted parasite proliferation. These findings underscore the complex interplay between autophagy and tick-borne pathogens, highlighting its dual role as an immune effector and as a resource exploited by invading pathogens to support their survival and colonization within tick midguts.

Emerging evidence indicates that some pathogens concurrently induce autophagy and apoptosis in host cells to enhance their survival, replication, and transmission. For instance, *Toxoplasma gondii* secretes effector proteins such as ROP16 and ROP18, which modulate autophagosome formation, leading to the encapsulation of the parasite within a protective endo-vesicular structure, thereby facilitating immune evasion during early infection stages (Cheng et al., 2022). Additionally, *T. gondii* activates host caspase pathways, particularly *caspase-3*, which plays a critical role in inducing cell apoptosis, facilitating parasite egress and propagation (Payne et al., 2003). Similarly, *Plasmodium* spp. promotes parasite proliferation by activating autophagic pathways, enabling the acquisition of essential nutrients such as amino acids and lipids from host cells (Thieleke-Matos et al., 2016). It also induces apoptosis in erythrocytes to promote parasite release and systemic spread of the infection. Consistent with these findings, the present study demonstrated that *Babesia* infection induces both autophagy and apoptosis in ticks to increase infectivity. Functional validation through RNAi confirmed that silencing of *caspase-7, caspase-9*, and *ATG5* genes significantly reduced *B. microti* loads, reinforcing the hypothesis that these pathways are co-opted by the parasite to enhance its proliferation and survival within the tick midgut. While transcriptional changes in apoptosis (*caspase-7, caspase-9*) and autophagy (*ATG5, ATG8*) genes were observed, protein-level validation (cleaved caspases, LC3B-II) is required to confirm pathway activation. Future studies should quantify caspase cleavage and LC3-I/II conversion via Western blot to elucidate their roles in *Babesia*-tick interactions. Future investigations should examine the incidence of these processes in both infected and adjacent uninfected cells, characterize their cell-type specificity (e.g., differentiating between immune and non-immune populations), and determine the relative contributions of apoptosis and autophagy to the course of *B. microti* infection. Clarifying these spatial and cellular dynamics will elucidate how *Babesia* modulates host pathways, informing novel intervention strategies. Subsequent studies are warranted to elucidate the precise molecular mechanism underlying the modulatory role of *B. microti* on autophagy and apoptosis in ticks and to provide crucial insights into the cellular pathogenesis of *Babesia* infection, establishing a theoretical foundation for novel intervention strategies.

GO and KEGG enrichment analyses suggest that *B. microti* infection may modulate autophagy and apoptosis pathways in the tick midgut. Subsequent experimental validation confirmed that both processes facilitate *B. microti* infection. Collectively, these findings provide robust molecular evidence that *B. microti* actively modulates tick autophagy and apoptosis pathways to promote its survival and vector competence, offering novel insights into the complex interactions between *B. microti* and its tick vector, *H. longicornis*.

## 6 Conclusion

This study demonstrated that *B. microti* infection significantly upregulated genes associated with PCD pathways, particularly apoptosis and autophagy, in *H. longicornis*. Functional experiments demonstrated that RNAi-mediated knockdown of *caspase-7, caspase-9*, and *ATG5* genes effectively suppressed parasite proliferation, highlighting the pro-parasitic roles of apoptosis and autophagy in *B. microti*-infected ticks. These findings strongly suggest that *B. microti* modulates host PCD mechanisms to enhance its survival and transmission potential in tick midguts. Furthermore, this study provides a theoretical foundation for future investigations into the precise molecular mechanisms underlying *Babesia*-tick interactions and highlights the potential of targeting apoptosis and autophagy pathways as transmission-blocking strategies against tick-borne babesiosis.

## Data Availability

The raw sequencing data supporting the findings of this study have been deposited in the NCBI Sequence Read Archive (SRA) under the BioProject accession number PRJNA1328440.
