# Supplementary material for: Apoptosis and autophagy promote Babesia microti infection in tick midguts: insights from transcriptomic and functional RNAi studies
Source: Front Microbiol. 2025 Sep 19;16:1632974. doi: 10.3389/fmicb.2025.1632974 (PMC12491973; doi:10.3389/fmicb.2025.1632974)
Supplement: Supplementary file 1 [file Table_1.docx]

**Supplementary Table S1** The primers of detection of *B. microti*

| **Gene name** | **Primer sequence (5’-3’)** | **Amplicon size (bp)** |
| --- | --- | --- |
| *B.* *microti*-1-F: | AATTACCCAATCCTGACACAGG | 485 |
| *B. microti*-1-R: | TTTCGCAGTAGTTCGTCTTTAACA |  |
| *B. microti*-2-F: | GACACAGGGAGGTAGTGACAAGA | 407 |
| *B. microti*-2-R: | CCCAACTGCTCCTATTAACCATTAC |  |
| *B.microti* -qF: | AACAGGCATTCGCCTTGAAT | 104 |
| *B.microti* -qR: | CCAACTGCTCCTATTAACCATTACTCT |  |
| Probe | FAM-CTACAGCATGGAATAATGA-MGB |  |
