# Supplementary material for: Apoptosis and autophagy promote Babesia microti infection in tick midguts: insights from transcriptomic and functional RNAi studies
Source: Front Microbiol. 2025 Sep 19;16:1632974. doi: 10.3389/fmicb.2025.1632974 (PMC12491973; doi:10.3389/fmicb.2025.1632974)
Supplement: Supplementary file 2 [file Table_2.docx]

**Supplementary Table S2** The primers of qPCR

| **Gene name** | **Primer sequence (5’-3’)** | **Amplicon size** **(bp)** |
| --- | --- | --- |
| HL*Caspase-7*-qF: | TGCGGGATACGGATGAAACG | 135 |
| HL*Caspase-7*-qR: | GCATAACCATTGGCAGCGATA |  |
| HL*Caspase-8*-qF | ATGTCGGCGTACAAAGC | 87 |
| HL*Caspase-8*-qR | CTGTCCACCGCATCTCG |  |
| HL*Caspase-9*-qF: | GACGGAAGTCGGACATTG | 115 |
| HL*Caspase-9*-qR: | TCCAACCAGGAAGCCAC |  |
| HL*ATG5*-qF: | AACCAGCTGTGGATAGGAATG | 112 |
| HL*ATG5*-qR: | GCCGGAAAGGAATGTGTTTG |  |
| HL*ATG6*-qF: | TTCAAGTTCCTGTGGGACAC | 102 |
| HL*ATG6*-qR: | GAAGTTCGAGTCCATCTTGCT |  |
| HL*ATG8*-qF: | GAAGGCTGAAGACGGCATAC | 122 |
| HL*ATG8*-qR: | GGTATTCGCTGCGGGTATTT |  |
| ELF1A-qF： | CGTCTACAAGATTGGTGGCATT | 106 |
| ELF1A-qR： | CTCAGTGGTCAGGTTGGCAG |  |
