# Supplementary material for: Apoptosis and autophagy promote Babesia microti infection in tick midguts: insights from transcriptomic and functional RNAi studies
Source: Front Microbiol. 2025 Sep 19;16:1632974. doi: 10.3389/fmicb.2025.1632974 (PMC12491973; doi:10.3389/fmicb.2025.1632974)
Supplement: Supplementary file 3 [file Table_3.docx]

**Supplementary Table** **S3** The primers of dsRNA

| **Gene name** | **Primer sequence (5’-3’)** | **Amplicon size (bp)** |
| --- | --- | --- |
| HL*Caspase-7* dsRNA-S1: | GGATCCTAATACGACTCACTATAGG  GAAGTATCGCTGCCAATG | 448 |
| HL*Caspase-7* dsRNA-A1: | CGTCAGTTCCGTTATCCC |  |
| HL*Caspase-7* dsRNA-S2: | GAAGTATCGCTGCCAATG |  |
| HL*Caspase-7* dsRNA-A2: | GGATCCTAATACGACTCACTATAGG  CGTCAGTTCCGTTATCCC |  |
| HL*Caspase-9* dsRNA-S1: | GGATCCTAATACGACTCACTATAGG  GTTTGCGAGAACGAGGTG | 449 |
| HL*Caspase-9* dsRNA-A1: | TGCAGTGAGGTTAAGGTGG |  |
| HL*Caspase-9* dsRNA-S2: | GTTTGCGAGAACGAGGTG |  |
| HL*Caspase-9* dsRNA-A2: | GGATCCTAATACGACTCACTATAGG  TGCAGTGAGGTTAAGGTGG |  |
| HL*ATG5* dsRNA-S1: | GGATCCTAATACGACTCACTATAGGATGGCAGAAGATAGGGAAGTAC | 435 |
| HL*ATG5* dsRNA-A1: | GCCAACCACTTGACTGC |  |
| HL*ATG5* dsRNA-S2: | ATGGCAGAAGATAGGGAAGTAC |  |
| HL*ATG5* dsRNA-A2: | GGATCCTAATACGACTCACTATAGG GCCAACCACTTGACTGC |  |
| Luciferase dsRNA-S1: | GGATCCTAATACGACTCACTATAGG  GCTTCCATCTTCCAGGGATAC | 294 |
| Luciferase dsRNA-A1： | CGTCCACAAACACAACTCCTCC |  |
| Luciferase dsRNA-S2： | GCTTCCATCTTCCAGGGATACG |  |
| Luciferase dsRNA-A2： | GGATCCTAATACGACTCACTATAGG  CGTCCACAAACACAACTCCTC |  |

Note: The horizontal line below the primer represents the joined T7 promoter sequence.
