# Supplementary material for: Apoptosis and autophagy promote Babesia microti infection in tick midguts: insights from transcriptomic and functional RNAi studies
Source: Front Microbiol. 2025 Sep 19;16:1632974. doi: 10.3389/fmicb.2025.1632974 (PMC12491973; doi:10.3389/fmicb.2025.1632974)
Supplement: Supplementary file 6 [file Data_Sheet_1.docx]

**
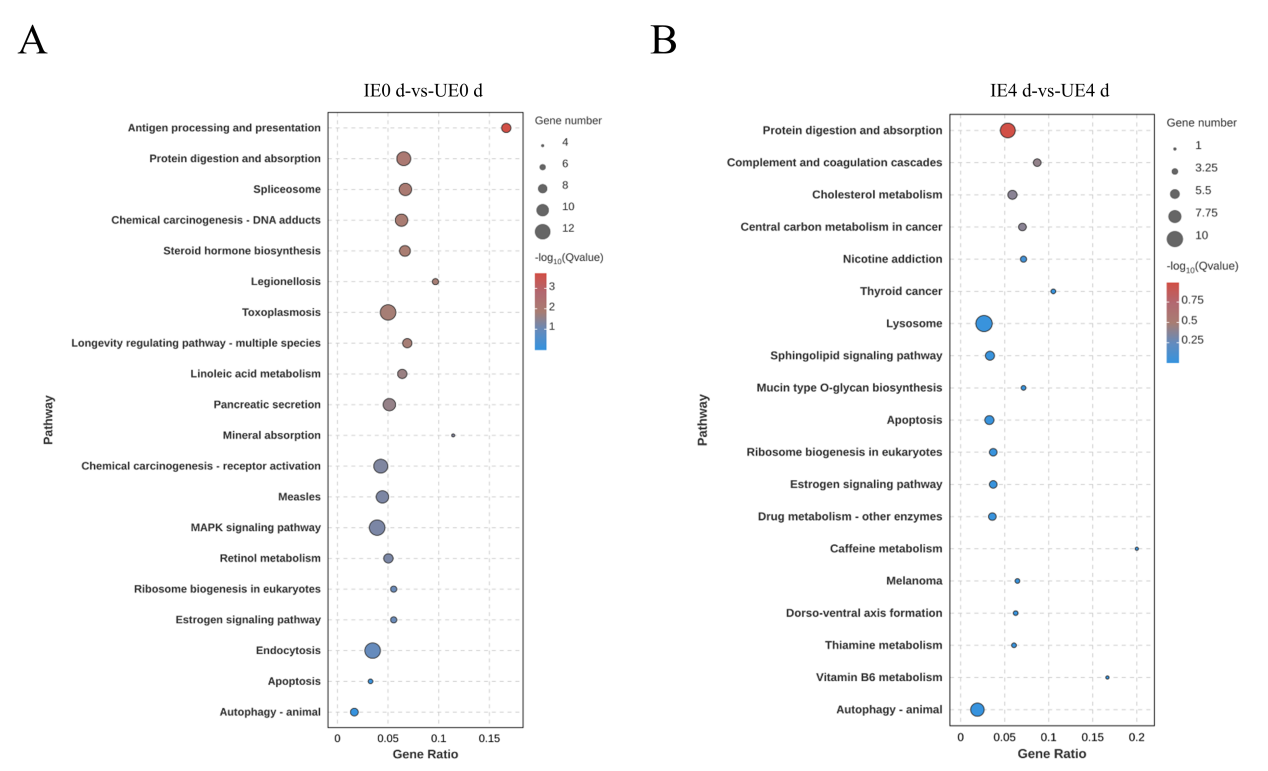
**

**FIGURE 1. Scatter plots of KEGG pathway enrichment of DEGs in *B. microti-*infected tick midguts at** (**A**) 0 d and (**B**) 4 days post-engorgement. The x-axis represents the rich factor (ratio of DEGs annotated to a pathway to the total genes in that pathway), while the y-axis represents significantly enriched KEGG pathways. Dot colors denote the enrichment score [-log1o(p-value)], with blue indicating low enrichment and red indicating high enrichment. Dot size represents the number of DEGs associated with the corresponding KEGG pathway, with bigger dots indicating a larger number of DEGs. IE0 d: Infected *B. microti* at 0 day post-engorgement; IE4 d: Infected *B. microti* at 4 days post-engorgement; UE0 d: Uninfected *B. microti* at 0 day post-engorgement; UE4 d: Uninfected *B. microti* at 4 days post-engorgement.
