# Supplementary figures and images for: Apoptosis and autophagy promote Babesia microti infection in tick midguts: insights from transcriptomic and functional RNAi studies
Source: Front Microbiol. 2025 Sep 19;16:1632974. doi: 10.3389/fmicb.2025.1632974 (PMC12491973; doi:10.3389/fmicb.2025.1632974)

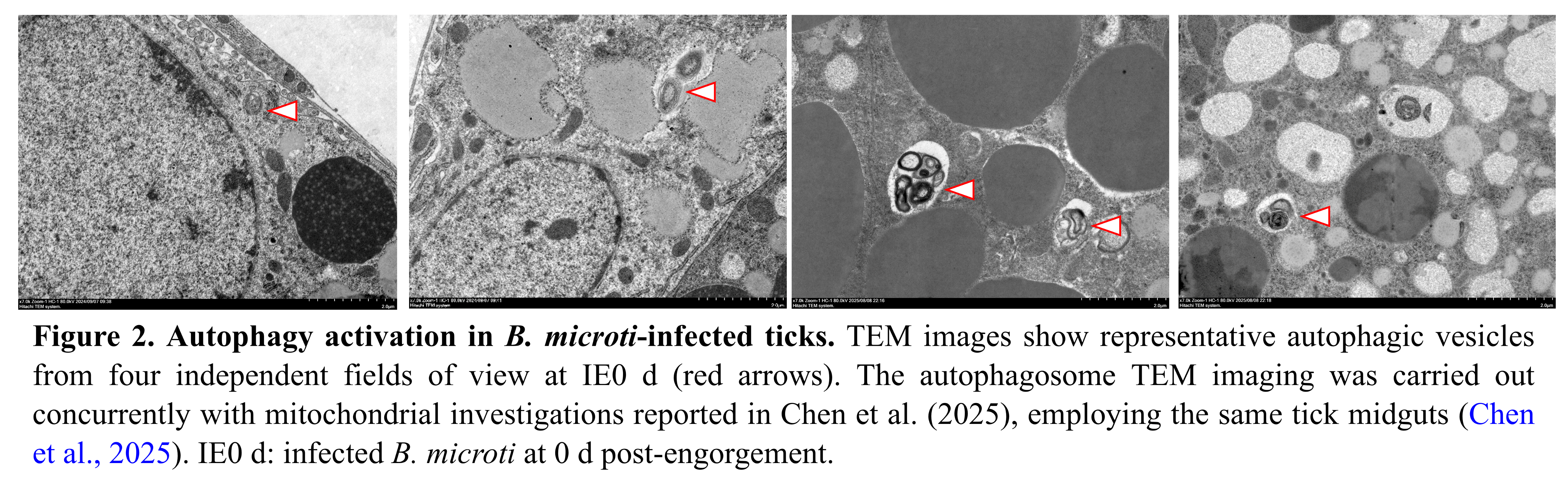

Supplement: Supplementary file 7 [file Image_1.tif]
